# Employing Yarning to Explore the Impact of Endorsed Midwives in an Aboriginal Medical Service

**DOI:** 10.1002/nop2.70357

**Published:** 2025-10-30

**Authors:** Assimina Di Lollo, Susan Smith, Tahlia Johnson, Nina Sivertsen

**Affiliations:** ^1^ College of Nursing and Health Sciences, Caring Futures Institute Flinders University Adelaide South Australia Australia; ^2^ Faculty of Health Sciences UiT Arctic University of Norway Tromsø Norway

**Keywords:** Aboriginal Community‐Controlled Health Organisation, Aboriginal maternal health, Aboriginal Medical Service, endorsed midwife

## Abstract

**Aim:**

The aim of this study was to explore the role of Endorsed Midwives employed in an Aboriginal Medical Service (AMS) and to assess the impact on Aboriginal mothers and babies using the service.

**Background:**

Current research on the role of the Endorsed Midwife (EM) is limited, with no available evidence regarding its application in an AMS setting. Health disparities persist in the outcomes of Aboriginal and non‐Aboriginal mothers and babies. Aboriginal Community‐Controlled Health Organisation (ACCHO) strives to address these challenges by providing culturally safe maternity care. However, there is minimal evidence on the effectiveness of EMs in AMS settings.

**Method:**

This study used yarning to conduct interviews with EMs and Yorgas receiving care in an AMS in Western Australia.

**Results:**

Key themes identified from the EMs included greater job satisfaction, increased flexibility to deliver holistic care and enhanced collaboration within the multidisciplinary team. In contrast, the Yorgas emphasised the development of trusting relationships with the EMs and their appreciation for culturally appropriate, continuous care.

**Conclusions:**

This study provides a holistic perspective on the role of EMs in AMS settings, contributing valuable knowledge to EMs' practice while amplifying the voices of Yorgas who accessed the AMS.

**Implications for the Profession:**

The results of this study indicate that EMs possess enhanced capacity and flexibility in their roles, which contributes to greater job satisfaction whilst better meeting the needs of Yorgas' birthing in AMSs.

**Impact:**

This study explored the impact of Endorsed Midwives in an AMS. Endorsed midwives achieved greater job satisfaction and were well received by Aboriginal patients. This research increased knowledge of the benefits of Endorsed Midwives employed in an AMS, thereby enhancing the care of Aboriginal clients.

**Reporting Method:**

This study adhered to the SQRQ guidelines for reporting qualitative research.

**Patient/Public Contribution:**

The study was overseen by an Aboriginal advisory circle, who actively participated in the research process with a focus on culturally appropriate ways of knowing, being and doing research. The Advisory circle was involved from conception and design to data acquisition, analysis, as well as interpretation of data.

## 1 Introduction

In Australia, Aboriginal Medical Services (AMS's) and Aboriginal Community Controlled Health organisations (ACCHO's) both deliver culturally appropriate healthcare to Aboriginal and Torres Strait Islander peoples. Though often used interchangeably, they are distinct in governance and structure. AMSs are a broad category of services that may be government‐run or community‐led, depending on their location, history and operations (Australian College of Rural and Remote Medicine (ACCRRM) 2025). ACCHOs, by contrast, are always community‐initiated, governed and operated, with elected boards accountable to the local Aboriginal community. They are founded on principles of self‐determination and cultural safety (National Aboriginal Community Controlled Health Organisation (NACCHO) 2025a, 2025b).

The core difference lies in accountability: while some AMSs are community‐controlled, ACCHOs always are. Despite structural differences, AMSs and ACCHOs often collaborate on advocacy, service delivery and policy, especially through national bodies like NACCHO (NACCHO, NACCHO 2025a, 2025b), contributing to improved health outcomes for Aboriginal and Torres Strait Islander peoples.

Evidence suggests that Aboriginal and Torres Strait Islander women (Yorgas) are less likely to engage with mainstream maternity services and more likely to engage with culturally safe maternity services (McCalman et al. 2023). Aboriginal Community‐Controlled Health Organisation (ACCHO) proactively works towards closing the gap in health disparities between Aboriginal and non‐Aboriginal mothers and babies in Australia by providing culturally safe maternity services (Bertilone and McEvoy 2015; Cousins 2023; Kildea et al. 2018).

In Australia, Aboriginal Medical Services (AMS's) and Aboriginal Community Controlled Health Organisations (ACCHO's) both deliver culturally appropriate healthcare to Aboriginal and Torres Strait Islander peoples. Though often used interchangeably, they are distinct in governance and structure. AMSs are a broad category of services that may be government‐run or community‐led, depending on their location, history and operations (Australian College of Rural and Remote Medicine [ACCRRM] 2025). ACCHOs, by contrast, are always community‐initiated, governed and operated, with elected boards accountable to the local Aboriginal community. They are founded on principles of self‐determination and cultural safety (National Aboriginal Community Controlled Health Organisation [NACCHO] 2025a, 2025b). The core difference lies in accountability: while some AMSs are community‐controlled, ACCHOs always are. Despite structural differences, AMSs and ACCHOs often collaborate on advocacy, service delivery and policy, especially through national bodies like NACCHO (NACCHO 2025a, 2025b), contributing to improved health outcomes for Aboriginal and Torres Strait Islander peoples.

ACCHO's aim is to provide culturally safe care by responding at a grassroots level to the social determinants of health affecting Aboriginal people. Their philosophy is based on a social context of health as well as Aboriginal views of health which are often divergent from mainstream health providers (Khoury 2015). Hence the importance of understanding what Yorgas want from maternity services. This enables the services to provide evidence‐based practice, encourage self‐determination and value Aboriginal health knowledge (Marriott et al. 2019; McCalman et al. 2023).

The Australian Government identified three important areas for developing maternity services for Yorgas (Australian Government Department of Health and Ageing 2019). These included: increasing the Aboriginal healthcare workforce; increasing culturally competent care; and developing dedicated programmes for birthing on country (Kildea et al. 2018; Cousins 2023). In 2022 there were 6500 midwives with midwife‐only registration, and 28,800 midwives with dual midwife and registered nurse registrations in Australia. Of those registered midwives, only 908 held endorsement for scheduled medicines in Australia. Anecdotally we know that very few of these endorsed midwives (EM) work across the 144 AMSs in Australia. The Australian Institute of Health and Welfare reports that data on the number of midwives currently employed in ACCHO is unavailable (Australian Government Australian Institure of health and Welfare 2023). EMs have advanced skills which may be well suited to enhance maternity care and provide better options for care to Yorgas in the AMS setting. Legislation has allowed advanced practice midwives to gain endorsement with AHPRA since 2010 (Nursing and Midwifery Board of Australia 2024) (Reeve et al. 2016). These advanced skills include the ability to prescribe scheduled medicine, order diagnostic tests including pathology and medical imaging in addition to their standard midwifery practices (Nursing and Midwifery Board of Australia 2024).

A literature review was conducted prior to the commencement of this study, which revealed minimal research on EMs practice. Additionally, no data was located that provided information on EMs practice in the AMS setting. Of the research, that was located, Small et al. (2016) found EMs experienced greater satisfaction and motivation in their role. The EM believed they had the ability to provide greater flexibility and access to care, provide access to timely appropriate medications and improve clients' satisfaction. Barriers to the role identified a lack of clinical governance and specific education for the role (Small et al. 2016). The study by Medway et al. (2021) identified restricted access to the Pharmaceutical Benefits Scheme (PBS) and Medicare Benefits Schedule (MBS) items, as well as a lack of understanding and support for the role outside the private practicing Midwifery setting as additional barriers. These identified barriers encouraged fewer midwives to seek endorsement. Additionally, the study by Watson et al. (2002) confirmed a lack of support for prescribing medication as a barrier to providing optimal care.

A recent scoping review identified that Endorsed Midwives increase women's access to prescribed medicines; however, there remains limited data available describing how having Endorsed Midwives in an AMS setting can enhance maternal care for Yorgas (Hull et al. 2024). Hence, the aim of this study is to explore the experiences of EMs in an AMS setting and to give voice to the Yorgas' experiences of maternity care provided by EMs in the AMS. The setting for this research is an AMS with four EMs working in the service as a team. Evidence from this study may be valuable to inform guidelines, policy and procedures for an improved model of care that could be replicated across other ATSICCH.

## 2 Methods

### 2.1 Study Design

This research was conducted in collaboration with an AMS responsible for providing maternity care to Aboriginal families in Western Australia. The study was overseen by an Aboriginal advisory circle, who actively participated in the research process with a focus on culturally appropriate ways of knowing, being and doing research. The Advisory circle was involved from conception and design to data acquisition, analysis, as well as interpretation of data.

This study adopted yarning to gain an in‐depth understanding of EM practice within the cultural context of the AMS (Asselin 2003; Bessarab and Ng'andu 2010; Kennedy et al. 2022; Marriott et al. 2021; Geia et al. 2013). Yarning is an Aboriginal style of conversation whereby stories and knowledge are shared in a relaxed informal way and is recognised as a culturally appropriate Aboriginal method of data collection (Bessarab and Ng'andu 2010; Lin et al. 2016). Yarning circle topic guides were developed for both EMs and Yorgas to guide the yarning and build on evidence obtained in the literature review.

Colonisation is widely acknowledged as a negative determinant of health for Aboriginal people, contributing to systemic inequities, intergenerational trauma and the erosion of cultural practices and autonomy (Churchill et al. 2020; Scott‐Jones and Watt 2010; Sivertsen et al. 2022). These impacts have created ongoing barriers to accessing equitable healthcare and achieving positive health outcomes for Aboriginal communities (Sivertsen et al. 2020). Recognising this, a decolonising approach was selected to underpin this qualitative study, ensuring that the research actively sought to challenge and dismantle colonial biases and practices within healthcare systems and research methodologies (Churchill et al. 2020; Scott‐Jones and Watt 2010).

A decolonising approach prioritises Aboriginal perspectives, knowledge systems and lived experiences, centring their voices and agency in the research process (Smith 2021). This required critically examining power dynamics, addressing historical injustices and ensuring the research avoided harm or inequities. The research team worked in close collaboration with the Aboriginal community; respect for cultural protocols, and cultural safety were central principles. In this study, a decolonising framework guided the use of yarning as a methodology, aligning with Aboriginal ways of knowing and sharing knowledge. It also shaped the analysis, ensuring findings reflected the priorities and values of Aboriginal participants. This approach aimed to promote culturally safe practices and address structural inequities in maternity care for Yorgas. This approach guided our inclusion of Aboriginal views of health, values, knowledge and incorporated the voices of Yorgas. A focus on the Yorgas' engagement with Aboriginal Health Practitioners (AHP) and Aboriginal Outreach Workers (AOW) was central to this study (Garces‐Ozanne et al. 2016).

### 2.2 Ethical Considerations

Careful consideration was given to the ethical conduct of this study, as the setting was within an Aboriginal community and involved pregnant Yorgas. The voices of the Yorgas participants were an essential inclusion in the study, particularly because none of the EMs at the AMS identified as Aboriginal. The women's contributions provided an opportunity for data triangulation, enhancing the study's rigour and credibility (Polit 2016). However, given the very small number of EMs employed in ACCHOs and participating in the study, it is possible that individuals/institutions with knowledge about the ACCHO workforce in Western Australia would be able to identify the EMs who participated in the study. For this reason, no further information has been included about participant characteristics.

To ensure cultural safety and ethical appropriateness, a letter of support from the Aboriginal Community Controlled Health Organisation (ACCHO) committee was sought and obtained before commencing the research. The study design was developed in collaboration with the ACCHO, ensuring it aligned with community priorities and upheld the principles of respect, reciprocity and community ownership.

Yarning, as a culturally appropriate methodology, was employed to create a safe and respectful environment for participants, allowing them to share their experiences comfortably. Informed consent was obtained in a culturally sensitive manner, with ample time provided for participants to consider their involvement, and all consent materials were written in plain language.

Adhering to the National Health and Medical Research Council's (NHMRC) *Guidelines for Ethical Conduct in Aboriginal and Torres Strait Islander Health Research*, the study prioritised the well‐being, voices and perspectives of Yorgas, with considerable effort made to ensure safe, respectful research that included and was of benefit to the Aboriginal community (NHMRC 2018).

### 2.3 Participants and Recruitment

**FIGURE 1:**
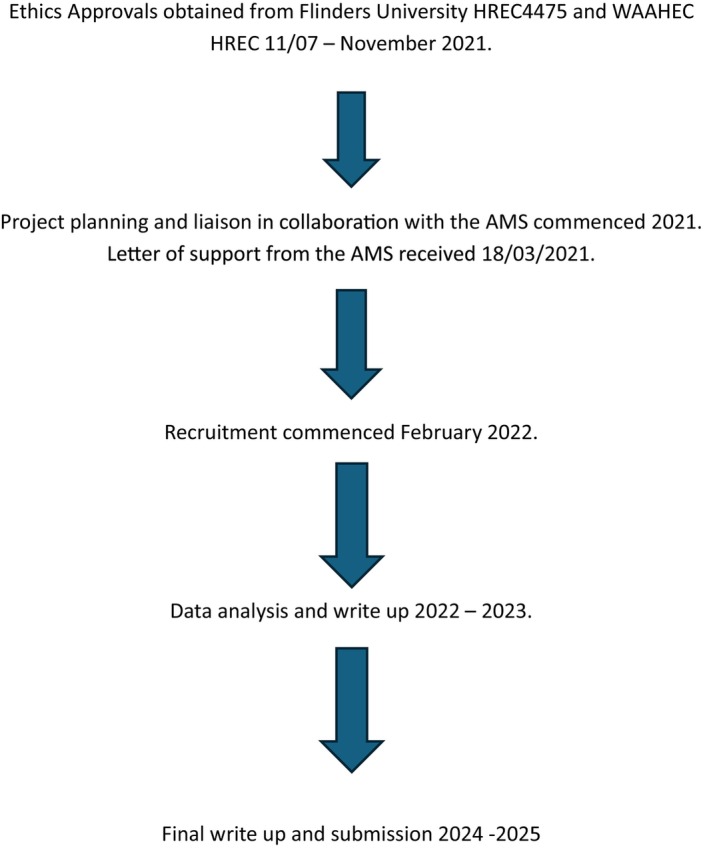
Recruitment timeline.

The participants in this study included EMs working in one AMS and Yorgas receiving maternity care at the same AMS. EMs and Yorgas using the service were invited to participate in the study. (See recruitment timeline at Figure 1). This research employed purposive sampling techniques to ensure the inclusion of participants most relevant to the study's objectives (Andrade 2021). The inclusion criteria required participants to be current maternity clients of the AMS, either pregnant or up to 6 weeks postpartum, and over 18 years of age. Exclusion criteria included those under 18 years of age or unable to provide informed consent. A total of 11 Yorgas were recruited, and three (*n* = 3) yarning circles were conducted. No financial incentives were provided to support recruitment.

#### 2.3.1 Endorsed Midwives (EMs)

The EMs recruited for the study were employed at the AMS and were invited to participate through email invitations sent by the practice manager. Three EMs (*n* = 3) employed at the AMS accepted the invitation and provided informed consent to participate. Two yarning circles were subsequently conducted to gather their insights and experiences as they were not all available at the same time due to work commitments.

#### 2.3.2 Yorgas

Flyers were created and displayed at the AMS prior to the commencement of the study. Information about the study was provided to Yorgas receiving maternity care by AHPs and AOWs. Women recruited during their clinical appointments were then randomly assigned to one of three yarning circles. The AHPs and AOWs facilitated the recruitment process and ensured fully informed written consent was obtained from all women who voluntarily agreed to participate in the yarning circles.

Recruitment of all participants (*n* = 14) and conduct of the yarning circle for Yorgas took place consecutively over a 6‐week period due to time constraints and availability of the setting. Additionally, recruitment to this study took place during a COVID‐19 outbreak in the community and this prevented some participation.

### 2.4 Data Collection

The yarning sessions were held in a family group room at an AMS maternal and child health clinic/house. This location was chosen for its privacy, familiarity and comfort, as it is regularly used for yarning sessions with Yorgas, facilitated by AHPs and AOWs. The setting was culturally appropriate and respectful, fostering a safe and welcoming environment for participants (Lin et al. 2016; Marriott et al. 2019).

Homogeneous yarning circles were organised to mitigate potential power imbalances between participant groups. Such grouping ensures participants share compatible experiences, which supports reliable data generation (Katz‐Buonincontro and Nezu 2022). It also promotes the exchange of ideas and the development of social consciousness, particularly among nurses with similar professional backgrounds (Thorne 2016).

The yarning sessions with EMs were facilitated by the first author, guided by a pre‐developed topic guide shared with participants beforehand. These sessions were capped at 60 min. Yarning circles with Yorgas were led by an AHP or AOW, with the first author present as a silent observer. These sessions lasted between 40 and 60 min. A topic guide was provided to the AHP and AOW facilitators prior to the sessions to ensure consistency and contained questions such as ‘tell me what is important to you when you come for Midwifery care’, ‘how do find accessing the midwifery care’ and ‘can you think of any ways we can improve or change the midwifery care’. All participants received an information sheet and provided written consent before participation. Yarning sessions were audio‐recorded with permission and securely stored on a password‐protected database, ensuring confidentiality and data integrity.

### 2.5 Data Analysis

Data obtained were transcribed verbatim and deidentified prior to coding. The transcribed data was verified by the AHP and AOW as an accurate reflection of yarning sessions. Analysis was undertaken as a team, with data inductively coded for content and meaning, then thematically analysed via a six‐phase method (Polit 2016). This was done initially by the Assimina Di Lollo, then cross‐checked and approved by the research team. Data were colour coded by hand with reference to yarning topics. Initial descriptive coding involved reading through qualitative data and coding passages that provided answers to the research questions. These were clustered according to emerging patterns. An interpretive second cycle involved filtering, highlighting and refining the meanings of codes into categories and concepts, then building analytical themes (Saldaña 2021). During this stage of iterative team discussion and synthesis, particular care was taken to view the findings through a decolonising lens (Sherwood and Anthony 2020). Themes arising from the coded data were then reviewed and subthemes identified and represented in tables (See Tables 1 and 2) (Clarke and Braun 2018). Any participant quotes used were anonymised to protect confidentiality.

**TABLE 1:** Endorsed midwife role yarning themes.

Theme	Subtheme	Subtheme	Description
Expectations of the role	Possibilities	Similar to UK role	See a huge difference in role and see the suitability in an AMS; role was huge before; requirements to become changed
			Like UK model of midwifery and similar autonomy
How E Midwives describe their role working to full potential	Facilitate, lead client care, Primary care giver	Provide timely individualised comprehensive care, function independently with assessment, investigation, order tests interpret results and initiate treatment	Sees role as being able to provide comprehensive care; ability to function independently; sees antenatal care as very important area of practice; believes as a primary care giver there is increased trust in the community; come straight to SWAMS when first pregnant; increased numbers; care about the women. Huge difference; although easier extra work
		Flexibility within performing role, time, location opportunistic care	Ability to provide individualised care to meet the needs of Yorgas (vit D, anaemia); more flexibility for opportunistic care (time, location); swift in delivery avoids multiple appointments and waiting for DRs; wholistic women centred care; builds relationships with clients; good to have access to MBS and PBS contributes to community; job satisfaction (love the role); improve care
	Benefits to organisation and clients	Access to MBS and PBS	More responsibility
Collaborator role		Scope of practice and confidenc	Very mindful of scope of practice and pathways for referral; equipped, confident to care for the normal to full scope of practice (good course); values/importance of the area of specialty.; can deal with what comes in
		Formal collaborative role developed pathway trust	Enabled by organisation into agreement, seen as a pathway not individually sought (AMS G.P.s; Hospital Obs. Regional G.P.O's). Understands that legal requirement. Believe Obs/G.P.s/G.P.O's trust value EM; Confident to reach out for advice/referral; Obs clinic; Assume greater responsibility as interpreting complex results for Obs/advocate for women. Greater responsibility as interpreting complex results and decision‐making working as advocate for women
		Formal Pathway part of MDT embedded into AMS	Very important recognises that is part of collaborative multidisciplinary team AHP/OW, allied health; mentor; values collaboration with other EM wrap around services
			EM's support/opinion/debrief
Cultural security cultural context	Indigenous view of health	Trusted caregiver	Believes appreciation; greater relationship building; confiding in midwives/confidentiality; important; debrief
		Wholistic care	Indigenous view of health; social emotional determinants Ability to meet needs, address physical emotional, social; guidance and support;(funerals) multifaceted role, SW, counsellor, transport; not always MO care led; closing the gap, wrap around services, setting
		Integral Team	AHP/OW gateway integral to access community
Enablers and barriers to the role	Enabled by	Organisation	Provider numbers organised by organisation for different locations; allows different locations for practice; allowing autonomy with timeframes and templates.
	Barriers to the role	Collaborative care	EM
		Specific education Reflective practice	Sees collaborative informal and formal pathway as enabler +EM hand over
		Awareness of the role	Lack of education P.D. opportunities specific to role; prescribing not practiced or confident; medicines limited
			No ongoing NPR, lack of self‐reflection
			Lack of understanding of the role

**TABLE 2:** Yorgas yarning themes.

Themes	Sub‐themes	Description
Valued elements of care	Motivating factors to come and use the AMS maternity service	Seek out a primary care giver; Comes for reassurance/confidence/looked after/hear baby/reduce fear
		Relaxed, chilled, family oriented, beautiful, comfortable, everyone's lovely
	Individualised care	Appreciate individualised care reminders and inclusion of AHP in the pathway book. Caters for the social emotional needs by making it easy otherwise wouldn't go
	Midwife role	Belief that the Midwives provide exceptional service above what might be normally experienced, go above and beyond
	Unsure of care pathway	An awareness of the care pathway not well understood by some women (lack of awareness of role?)
	Mainstream service comparison	Comparison to Hospital care. Describes hospital experiences as unpleasant, not individualised care depersonalised care, long waiting times, AMS service is comprehensive and do everything
Cultural safety	Individual needs/wholistic care	States that everyone has different needs and that the service Midwives meet those needs and do the best job
	Integrated service (collab)	Reduces anxiety to meet the Dr. in the AMS setting in a relaxed way and able to build a relationship with Dr. (result of collab)
	Level of cultural safety	Agrees culturally appropriate, but still not in their world, two worlds, happy for home visits, happy with AHP/OW, compared to mainstream poor, Karratha poor
Relationship	Therapeutic relationship building. Professional intimacy	Feel safe and can say/discuss anything; non‐judgemental; talk mate; trust decision making; Expect information/not hospital
	Trust	Reliable agrees will FU in own time, my midwife, wants through whole journey intrapartum; responsible do a good job
	Power	Advocate for the women, experiences at scans, build relationships Dr. as above in AMS setting
	Empathy	Involved in care understanding family needs
	Respect	Acknowledging individual needs and experiences/−ve Hospital judgemental cautious about/fear; answer individual questions
Continuity	Antenatal/postnatal	Important, loves MW, followed in care/only like seeing my midwife/better than different people/fu in own time
		Advocate role/FU after US experience bad
		Not sure of care pathway
	Intrapartum care	Wants intrapartum/no need to debrief or can/DR at birth organised valued/Dr. not at birth
		MGP good experience; 24/7 access; now experienced not needed
		MGP negative waiting times; bad had to make own appointments

## 3 Findings

Four key themes were identified from the data collected from Endorsed Midwives: (1) ‘working to full potential’; (2) collaboration is key to improved care experiences; (3) working towards culturally safe care; (4) enablers and barriers to best practice.

Similarly, four main themes were identified from the data provided by Yorgas: (1) valued elements of care; (2) ‘culturally appropriate, but still not in my world’; (3) ‘My midwife’– relationships; (4) and continuity of carer. All quotes from EMs labelled as M1–M3 and those from Yorgas labelled as Y1–Y11. Quotes highlighting the themes are presented in italics and indented to clearly signify participant voices.

### 3.1 Key Themes EMs

#### 3.1.1 Theme 1: ‘Working to Full Potential’

The midwives who participated in this study were working in the AMS setting prior to undertaking further education to become EMs. All midwives held positive expectations of what the EM role might add to their practice. One EM acknowledged that her role working in the AMS was ‘hugely significant for the health of the women’, before becoming endorsed but thought an AMS was an ideal place to utilise the broader skill set (M2). M2 also described overcoming hurdles with the Nursing and Midwifery Board to be able to qualify as an EM. As a result of this negotiation, midwives seeking endorsement (EM) no longer need to be current in the birth suite. M1 had worked as a community midwife in the UK and believed becoming endorsed would add more autonomy to her role comparable to the UK model of midwifery that she had experienced. The dominant theme arising from the EM yarning was being able to work at full potential: ‘we can just do everything’ and are therefore more able to provide ‘comprehensive care’ (M1).

We can be quite flexible in the way we deliver the service… we can function independently, and it probably avoids multiple appointments. (M3)

All EM participants thought that antenatal care in the AMS was an important and valuable area of practice where they could make a difference. The midwives saw themselves as the trusted primary care givers in the community and said that the women came to the AMS in early pregnancy and that the numbers of women engaging had increased.

…the word in the community is getting out there that we do offer this comprehensive care. (M1)

The EM reported a huge difference in their role after becoming endorsed stating it had made it easier to do their work, although it had added extra workload and responsibility to follow up on investigations. Being Endorsed provided greater ability to plan and provide flexible individualised care to meet the needs of Yorgas along with the knowledge of issues affecting women in their community, such as low vitamin D, anaemia or the awareness of social issues impacting on care such as family crisis or funerals.

We often know the women the best out of all the care providers…. (M1)

…having the ability to think what our women need and have it just at our fingertips really. (M1)

By being Endorsed, midwives were more time efficient in their ability to deliver care, they had the ability to provide opportunistic care, using their advanced pathology and diagnostic skills in a variety of outreach locations without a medical doctor being present. Thereby, avoiding multiple appointments and waiting times to see a doctor for routine screening and diagnostic tests.

…you can just get on and do it and that is very satisfying. (M2)

… providing that culturally safe care, continuity of care, woman centred care, we can be quite flexible in the way we deliver the service…. home visits are potentially needed, flexible in the time that we see the ladies and we can function independently, it probably avoids multiple appointments …. I think it allows more flexibility in delivering care. (M1)

Two of the midwives described an increased ability to build relationships with the women and provide wholistic, women centred care. Additionally, one participant stated that they loved their role as an EM which provided increased job satisfaction and improved the care they provided.

We have the skills and the knowledge…and it's just working to our full capacity to be able to really improve the care we're giving and um with Aboriginal health we sometimes have to be opportunistic and so it enables that, to be able to have the ability to order the tests and anything we need to do for that lady, yeah, I find it quite rewarding. (M3)

Two participants believed it beneficial to have access to MBS; and Pharmaceutical Benefits Scheme in their extended role of being able to order diagnostic tests and prescribe which enabled them to give comprehensive care to the women and to give back into the AMS community.

…having access to the pharmaceutical benefits scheme is really great and the medical benefits scheme… so that's what's probably what's really changed. (M3)

This view was shared by multiple EM participants.

The EM role was reported as adding to their effectiveness and over all job satisfaction whilst better meeting the needs of the community. The EM believed that their greater skills and knowledge as well as their ability to prescribe, enhanced their efficiency and effectiveness as well as their job satisfaction. The midwives believed the university course they had undertaken to become eligible to be endorsed made them feel better equipped and more confident to care for women in a normal pregnancy and to deal with ‘whatever walked in the door’ (M1).

#### 3.1.2 Theme 2: Collaboration Is Key to Improved Care Experiences

All participants believed they were able to practice to their full scope of practice but were mindful of working within that scope. They described a good awareness of partnerships and the pathways for referral.

…being aware of what our scope of practice is and anything outside of that then, we will work still closely with other members of our multidisciplinary teams so our G.P.s, our health workers, our Aboriginal outreach workers …but if it's within scope then we can manage the pregnancy and then have good outcomes. (M3)

One participant stated that they valued their role in the AMS setting and that they believed it was an important area of specialty.

It's a very specialised area …we really do carry the maternity list, like the G.P.s are really not involved…you can go and ask them anything and we can go to the Obstetricians now but between us (Endorsed Midwives) we are very skilled. (M1)

All EMs stated that they were aware of the legal requirement to be within a formal collaborative agreement with either a general practice or hospital obstetric service to practice as an EM. They also saw this as being important to refer for specialist advice and care when outside of their scope of practice.

There will be times when we need to be able to refer or on, we need more specialist advice or care for the women, so I think it is good to have a collaborative relationship. (M3)

Obtaining the formal collaborative agreement was seen as a process that was enabled by the organisation, the process was not individually sought nor was it well understood. However, because of the agreement, the midwives assumed primary caregiver role for the Yorgas at the AMS. The midwives work closely with Obstetricians from the Regional Hospital as the AMS has no GPO. The Obstetricians come to the AMS clinic and saw the women with the EM, instead of attending the hospital clinic. The participants stated that attendance rate to Obstetrician appointments and follow up for non‐attendance has improved because of this.

One midwife believed that the Obstetricians, General practitioners and General Practice Obstetricians trusted and valued the EMs, and the midwives felt confident to reach out for advice or referral to the Obstetricians and General Practice Obstetricians.

We especially work well collaboratively, especially as we do the (Obstetricians) clinic now… I think we've all got their trust…. (M1)

Another important aspect of the role was working collaboratively as a mentor within a multidisciplinary team at the AMS, with AHPs and AOW and other allied health services. One EM stated they valued this collaboration with the wrap around services to be able to meet the needs of Yorgas in partnership with midwifery care.

…the wrap around service offered is what makes the service what it is, and it makes clients want to come here because we have the wrap‐around services…. (M2)

The midwives identified that their enhanced role and increased collaboration across the multidisciplinary team resulted in an overall improvement in care for the Yorgas in the community.

#### 3.1.3 Theme 3: Working Towards Culturally Safe Care

The midwives saw their role had become increasingly multifaceted not always having a midwifery focus, but sometimes taking a role of counsellor, transport officer or social worker. Midwives reported building trusting relationships with the women they cared for who often confided in them and appreciated their confidentiality; this was identified as an important aspect of the success of their role. They also described their awareness of the complex social and emotional determinants impacting Yorgas' health and felt that the EM role increased their ability to tailor the woman's care around those needs. For example, one EM described the importance of responding to circumstances as they presented.

… this woman is homeless and that, but she needs a GTT [Glucose tolerance test] … so, we must prioritise … being sensitive to what's needed at that time, and I think that we absolutely do it and we can do it. (M2)

By collaborating across the multidisciplinary team, rescheduling care if a woman had to travel to a funeral or finding a refuge for a woman escaping domestic violence the EM were able to provide culturally safe care.

The midwives also saw their role as making referrals to the AMS wrap‐around services, as well as to other agencies addressing social and emotional needs and medical needs.

… you have to be flexible to be able to provide that sensitive, culturally sensitive care, understand their priorities …like they have to get to Perth for a funeral I think you just have to go ok yep. (M2)

The participants saw the collaborative role between themselves and the AHP/AOW was a gateway into the community and an integral component for them to be able to access the community.

…if we didn't have our Aboriginal workers, we would really be nothing, because they are often our little link in to the women especially the difficult women, I don't mean difficult, I mean, complex women who don't always want to come in and see us or they have other things going on like and it's often that link in the community that to help us. (M1)

The level of culturally safe care provided by the EM was continually expanding as their role took on a new responsiveness to the needs of the community.

#### 3.1.4 Theme 4: Enablers and Barriers to Best Practice

The EMs all felt supported by the organisation which had enabled them to perform their role. The AMS obtained the EM provider numbers for multiple clinic locations, allowing greater autonomy to practice in different settings or communities. Additionally, the EMs had greater autonomy to arrange timeframes and templates at multiple locations increasing service access for women.

…it made a huge difference to my role not having to ask a G.P. to sign it off…

The formal collaborative role with the general practitioners, general practice obstetricians and obstetricians in the woman's care pathway was a valued aspect and aided access to appropriate services to meet the needs of the women.

…doing the obstetric clinic, it is hard work and is extra work, but the benefit far outweighs the work it costs me because we all love our job so much and you just want the best for the women and that is the best for the women. (M1)

EMs identified the informal collaborative role with AHP, AOW and the AMS allied services provided peer support and cultural knowledge to identify appropriate care.

Regular EM team collaboration was identified as an important process to provide peer support, share information and an opportunity to debrief.

…you two are definitely a great strength and support at work. (M1)

One participant identified the difficulty in completing continuous professional development specific to the EM role as there is limited access to training specific EM practice.

…we do have to keep our [continuous professional development] our professional development up so …having some more specific education. (M2)

Another area of deficit identified was a lack of ongoing practice review which could provide the opportunity for self‐reflection. For example, one midwife identified a lack of education particularly to prescribing. This was identified as a barrier to their role and identified a lack of confidence in prescribing.

I'm not confident with it (prescribing). (M1)

I would love to have some more professional development around that (prescribing). (M1)

One midwife identified that the list of medicines they were able to prescribe was too limited in comparison with nurse practitioners.

They are quite limited the medications … we are not in labour ward …what our Nurse Practitioner can prescribe is a lot, lot more than what we can… I thought I'd be prescribing more… The only thing I really do is antibiotics for Urinary Tract Infections. (M2)

Hence enablers and barriers were identified including a lack of awareness and understanding of the EM role within the community was identified as being a barrier to performing their role, whilst the increased autonomy in the role was a definite enabler.

### 3.2 Key Themes‐ Yorgas Yarning

The themes identified from the voices of the Yorgas who received care from the EMs emphasised the significance of continuity and trust in their relationship with their caregiver. The key themes included: *valued elements of care provided*, ‘culturally appropriate, but still not in my world’, ‘my midwife’ and continuity of care. These themes highlight the women's perspectives on the quality and cultural relevance of the care they received, as well as the importance of strong, consistent caregiver relationships.

#### 3.2.1 Theme 1: Valued Elements of Care

Most women stated they came to the EM at the AMS for reassurance that their baby was well and healthy and to hear the baby's heartbeat. They also came for information which they believed and trusted to be accurate.

… it's probably just that caring consideration the informative side of it … informed of the right information …is something I consider in high regard…. (Y1)

The women made many positive comments which suggested that they were satisfied with the service they received. It was described as comfortable, relaxed and family oriented and the EMs were described as ‘lovely’ and ‘caring’ and described the service as ‘beautiful’(Y1).

Many of the women reported that they appreciated individualised care from the EMS, including reminders for their appointments, booking scans and blood tests organising transport. Having individualised care made it ‘easy’ for them; otherwise they ‘wouldn't go’ to their appointments.

… I would probably not go to my appointments without that little bit of support in the background. (Y1)

The women commented that the EMs provide an exceptional service and went ‘above and beyond’ and can ‘can do everything’ in comparison to other services. Overall, the care was highly rated and valued by the participating women.

#### 3.2.2 Theme 2: ‘Culturally Appropriate, but Still not in in My World’

The Yorgas who participated in this research generally agreed that each person has individual needs, and that the AMS was able to meet those needs. The women were happy to receive home visits from the EM and the AHP/OW. They expressed gratitude in having people from the community (AHP/OW) caring for them whilst reflecting on the poor care previously received from mainstream services.

‘… I found that the hospitals were very judgemental, very judgemental…. I have come in here a few times, and I have seen AOW and AHP both being nieces of mine, but you know it's sort of like you're more relaxed you're more … Its more family orientated…. (Y3)

The AMS facilitates a clinic with the EM supporting the visiting Regional Hospital Obstetrician. The women commented positively that it made them more comfortable having a known EM with them. Additionally, the women were happy that the Hospital Obstetrician was offering a service at the AMS, commenting that it made it more comfortable and relaxed.

…when we go here it's really chilled out, relaxed not waiting hours and hours, so that's definitely a lot better. (Y9)

This clinic also helped to break down some of the negative beliefs about hospital care. The clinic which was held at the AMS reduced anxiety making it possible to build a relationship with an Obstetrician. The women felt as though they were receiving personalised care and felt they were not ‘just a number’.

…when I met the doctor, she was really lovely…. (Y9)

One participant commented on their experience of care from a hospital based Aboriginal Midwifery Group Practice (MGP) model and stated the EM AMS care was more relaxed due to not having to attend the hospital for any routine appointments.

The women stated they agreed they felt the service was culturally appropriate, but it was still not in their world, commenting that there were ‘two worlds’.

…because it's just two different worlds. (Y1)

The participants valued the experiences at the AMS, they felt culturally safe, comfortable, cared for and experienced less anxiety in that space, and whilst the AMS came close to ‘their world’ it remained somewhat alien to them.

#### 3.2.3 Theme 3: ‘My Midwife’—Relationships

An important theme that arose from the Yarning sessions was the strong therapeutic relationship that had formed between the EMs and the women.

Women reported they felt safe and were able to discuss anything and thought the EM was non‐judgmental.

… not be judged or at least be understood on why you're feeling the way you're feeling…. (Y2)

The women trusted the decision making of the EMs and the information they received and commented on the lack of information received from the Hospital.

… someone who is well informed in their role …they know what they are talking about … I put a lot of trust in that you know…. (Y1)

The women stated they believed the EMs were reliable and commented that many EMs were following up in their own time making them feel personally cared for.

…to form a friendship or a bond…here as opposed to anywhere up in Perth is beneficial, you get the chance and opportunity whereas up there you are practically a number, you get out of there as quick as you can. (Y2)

The women described the EM as ‘my midwife’ and some commented that they wanted their midwife to follow them through the ‘whole journey’ intrapartum.

… I don't really feel like a patient when I go there, I feel like I'm talking with a mate. (Y7)

The women recounted instances where the EMs advocated for them; a recurring example was unpleasant treatment when attending scans, upon reporting this to the EMs, formal complaints were lodged. The EMs being present during Obstetricians appointments helped to build relationships between the Obstetrician and the women.

… I love that it's just so good like having the Dr come to you guys make me feel so comfortable. (Y7)

The women felt there was an understanding of their family needs because of the close relationship between EM and Aboriginal Health Worker team, the health workers being from the community and often related or friends of clients.

The women stated they felt respected and acknowledged for their individual needs and experiences and the midwives were able to answer their individual questions.

…Definitely respect and integrity I'm feeling…. (Y1)

Whereas the women described how they felt judged, cautious and fearful when at the Hospital.

Overall, the participants expressed a sense of familiarity and support when attending the AMS. They felt safe and had a feeling of belonging as opposed to feelings of distrust when attending mainstream hospital facilities.

#### 3.2.4 Theme 4: Continuity

The women spoke about their experiences of antenatal and postnatal care.

All the participants spoke about the EMs who had cared for them by their first names and expressed positive regard for them and that they preferred to see their ‘own’ midwife and they valued this continuity.

… I only like seeing my midwife…. (Y10)

…I had to do the NIPT (Non‐invasive prenatal testing‐ a blood test which screen for chromosomal abnormalities) …I was up in Karratha at the time … EM was even messaging me and calling me in her own free time to check up how I was … she's nice…. (Y11)

Although the women were satisfied with the service, having an on‐call midwife was discussed from the MGP experience of one participant in one group as being something that the women might want.

… that would make me feel way more secure like I have that more need I don't know how to say it. (Y9)

Some confusion around the role of the EMs was evident, women commented that they had not understood the role of the EM well and had expectations that the EM was on call for them.

… I don't have a midwife I could call if I needed to, I wasn't sure if I call you guys? Or come to you? Or am I to go to my GP or the hospital…. (Y9)

The women highlighted issues around their intrapartum care. The AMS does not deliver intrapartum care, the women generally attend the Regional Hospital for their labour. The women expressed they wanted a change in the service that they wanted the EM to follow them into the hospital and care for them during labour. Reasons being they trusted the EMs knowledge also they knew them, they felt more confident in the EMs care and wanted the opportunity to debrief following the birth experience with the midwife.

… knowing that none of you three are going to be there when I give birth that freaks me out a little bit… I told K like, mums going to go get her, yeah that to me should change. (Y4)

…you trust what she's saying, if … someone says; O we're going to take you for an emergency c section and you think, shit… but if it's someone like yourself or the other midwives you'd say, I feel like I trust that you would do what's exactly best for me and I absolutely must do that you know. (Y5)

The participants in this study expressed anxiety in transitioning to the regional hospital for their intrapartum care. They expressed a desire for that service to be offered in the AMS. This preference was related to a strong desire for continuity of care with their allocated EM following them for labour and delivery.

## 4 Discussion

The findings of this study suggest that the role of the EM is supported in the AMS setting and has played a pivotal role in facilitating interagency collaboration and providing continuity of care within a culturally appropriate model of practice. The themes identified from the EM yarning circles provided valuable insights into their clinical application of the role, areas for improvement and their experiences, such as increased job satisfaction and enhanced ability to meet the needs of Yorgas. These findings underline the complexities and opportunities of their role in providing maternity care within an AMS.

This study has also identified that the role of the EM in this AMS setting is a successful example of the Australian Government Maternity Models of Care. Additionally, the EM role has allowed greater accessibility to maternity care and improved services by navigating a care pathway for Yorgas across agencies within a culturally appropriate model. Similarly, the Nurse Practitioners’ role was identified as a lynchpin to the facilitation of client care and enhanced communication between clients and medical caregivers (Williamson et al. 2012). Additionally, it became evident that the existence of AMSs has facilitated improved access and choice in maternity care. The data obtained in this study have proved insightful by providing first‐hand experience of how EMS are working to meet the needs of Yorgas within a culturally safe model of care. Additionally, the voices of Yorgas who received care provided important feedback into achieving best practice in the AMS.

This research identified that a collaborative agreement is necessary for the EM to access a Medicare provider number (APHRA 2024). In the AMS setting, the referral pathway has resulted in greater interagency partnership between the AMS and the regional hospital obstetricians. Improving interagency collaboration is an important principle of the Birthing on Country (BoC) model and an incentive of closing the gap to improve access to care and build the Aboriginal community‐controlled sector (Kildea et al. 2021). Interagency collaboration has many benefits such as improved access and engagement with quality obstetric care in a culturally safe environment and is an example of a quality partnership (Kruske 2013).

The EMs in this study described their relationship with Obstetricians as supportive. Similarly, findings by Skinner and Foureur (2010) found that Midwife/Obstetricians relationships were supportive and facilitated continuity of care in Midwife‐led models of care in New Zealand. The factors identified that supported the EM model in the AMS were: the collaborative arrangement between Hospital and AMS; assisting EMs to obtain a provider number; embedding the EM within the AHP/AOW team; access to the other EM for peer support; wrap‐around services of AMS to meet the needs of women, thereby enabling wholistic care; and the willingness of the AMS G.P.s and Hospital Obstetricians to participate in integrating services to the AMS. Similar research findings affirmed that support for the EM role was critical to its success and must come from multiple sources (Medway et al. 2021).

The development of maternity services in ACCHOs is an important way forward to provide culturally competent maternity care and address the negative impact of colonisation on first nations birthing by returning control to those communities (Kildea et al. 2021). Dissatisfaction with mainstream hospital maternity services was a main theme from the Yorgas yarning circles. Mainstream health workers also recognise the hospital system lacks cultural safety, continuity and flexibility and calls for change. This theme is consistent also with other contemporary research which demonstrated that the health system does not acknowledge culturally safe care practices, lacks access to Aboriginal health care workers and found midwives struggle to define culturally safe care (Marriott et al. 2021, 2019).

The Yorgas in this study perceived the EMs at the AMS as their primary caregiver and a trusted part of the AMS team being led into the community by the AHW/AOW. The advanced practice role of the EM in this setting was found to be able to provide continuity and greater flexibility to tailor care for each woman, all being important elements of BoC principles (Australian College of Midwives 2017). The evidence from BoC models in an urban setting found better outcomes, improved integration of care, support for families and an increased capability of the non‐Aboriginal workforce (Kildea et al. 2021). The principles of BoC that allow for traditional practices such as supporting connection to country and land; a holistic view of health; valuing Aboriginal and non‐Aboriginal ways of knowing; and being culturally competent; are developed with the Aboriginal community; are all evident in the yarning themes of the AMS EM role and provide additional evidence to the work of Hickey et al. (2018).

Continuity of care in midwifery models in the primary health setting has shown improvements in outcomes for Yorgas (Homer et al. 2012; Sivertsen et al. 2020; West et al. 2016). Research suggests that continuity of care such as that in MGP results in improved outcomes for mothers and babies; unfortunately access to these models for Yorgas is extremely limited (Perriman et al. 2018). However, with the Yorgas' high expectations of maternity care and a stated preference for continuity of care, there is a need for the communities with an AMS to understand how the presence of EMS can enhance these needs.

The findings of this study confirm previous research by Small et al. (2016) that EMs enjoy greater satisfaction in their role as do their clients. The EMs in this setting believed they were enjoying working to full scope of practice and believed the role is suited to the AMS as it allows them greater flexibility to deliver individualised, comprehensive care in a variety of settings. thereby confirming their expectations that gaining endorsement would make a difference to their AMS practice. These findings contradict the work of Medway et al. (2021) who found that EMs believed they could not work to full potential and encountered barriers in support. This difference may be explained by the context of the service setting and levels of support offered to EMs in the AMS.

The EMs identified other EMs in this practice as a strength and valuable for peer support, which confirms the findings of Medway et al. (2021). The 2021 study found that EMs relied on peer support among their colleagues. Having access to the MBS was also seen as an asset in this setting, a way of contributing to the community. Additionally, Medway et al. (2021) suggested that the rebateable diagnostic services should be extended (Australian Government Department of Health and Ageing 2023). However, this aspect was not explored in this study.

This study identified several aspects of the role that need further development, and these aspects were identified consistently across the limited research of the EM role. Those issues were a lack of education specific to the role, mentoring, peer support and prescribing (Small et al. 2016). Additionally, a lack of awareness of the role of the EM was also found to be an issue in consultation with the Yorgas yarning circles and identified as an area for further development. Woo et al. (2020) also found a lack of awareness and understanding by clients of the Nurse Practitioners' role in clinical practice. Their research recommended providing education to the target population when introducing a new role. This approach was supported by Medway et al. (2021) who suggested that there was a need to raise public awareness of the role and thereby contribute to its success.

This study also identified important data on the barriers EMs experience at work. These included: the limitation to the list of medicines they can prescribe. The EMs believed that this list could be much broader given their role is in a primary health care environment. Additionally, the opportunity for role‐specific CPD must be enhanced with a focus on supporting the EMs’ education in prescribing (Small et al. 2016). Given the vital role that EMs play in providing culturally safe maternity care to Aboriginal women in a primary care environment, it is important that these barriers are addressed.

### 4.1 Limitations and Future Directions

This study was limited by the small number of EM participants; however, this reflects the overall small number of EMs working in AMS settings across Australia. As such, while the findings may not be generalisable to all AMS settings, they provide valuable insights into a niche area of practice that is underrepresented in the literature. Variations in resources and support across different communities and ACCHO further limit the applicability of these results.

Future directions include reviewing medications to be added to the PBS to ensure alignment with best practice and relevance to this setting. Additional research is also needed to investigate the sustainability of the EM role in other settings and to identify and develop specific educational requirements to support and maintain best practice within this specialised role.

## 5 Conclusion

The findings of this study highlight that EMs possess enhanced capacity and flexibility in their roles, contributing to greater job satisfaction. However, the study also identified a clear need for ongoing professional development and peer support tailored to the unique requirements of the role, aligning with findings from previous research.

The voices of Yorgas emphasised the value they placed on continuity of care and the trusting relationships they built with their EMs within a culturally appropriate space. The women also expressed a desire for greater continuity of care through the intrapartum period, reinforcing the importance of consistency in caregiver relationships.

These findings offer valuable insights for the local AMS, supporting quality improvement initiatives and the adoption of evidence‐based practices. Furthermore, the results can guide other AMSs considering the implementation of this model of care, contributing to its broader applicability and success in enhancing culturally safe maternity care.

## Author Contributions

Conception and design of the study by Assimina Di Lollo and Nina Sivertsen. Acquisition of data, yarning and interviews by Assimina Di Lollo. Analysis and interpretation of data by Assimina Di Lollo and Nina Sivertsen. Original draft of the article by Assimina Di Lollo. Critical revision of the manuscript for important intellectual content and analysis and interpretation of data by Nina Sivertsen, Tahlia Johnson, Susan Smith and Assimina Di Lollo. Final approval of the version to be submitted by Assimina Di Lollo, Susan Smith, Tahlia Johnson and Nina Sivertsen.

## Disclosure

This study uses the term ‘Yorgas’ to refer to Aboriginal women. ‘Yorga’ is a term used to describe an Aboriginal woman, originating from the South‐Western region of Western Australia (English Oxford Dictionary 2024).

In Australia, care must be taken to ensure appropriate terminology when referring to Aboriginal and Torres Strait Islander people and communities. This study has followed the Aboriginal and Torres Strait Islander guide to terminology endorsed by the Public Health Association of Australia. Please note that in this study the term Aboriginal is used; however we acknowledge that there is great diversity in the Aboriginal population (Australian Government—Australian Public Service Commission 2022).

## Ethics Statement

Ethical approval for this project was granted by the Flinders University Social and Behavioural Research Ethics Committee approval Project No. 4475, and Western Australia Aboriginal Health Ethics Committee Approval No. HREC1107.

## Conflicts of Interest

The authors declare no conflicts of interest.

## Data Availability

The data that support the findings of this study are available on request from the corresponding author. The data are not publicly available due to privacy or ethical restrictions.
